# The Importance of Subjectivity in Assessing the Efficiency of Surgery for Obstructive Sleep Apnea. Comment on Kuik et al. Subjective Efficiency Evaluation after Maxillomandibular Advancement Surgery in Obstructive Sleep Apnea Patients. *J. Clin. Med.* 2023, *12*, 4023

**DOI:** 10.3390/jcm12216761

**Published:** 2023-10-26

**Authors:** Antonino Maniaci, Salvatore Cocuzza, Giovanni Cammaroto, Claudio Vicini, Luigi La Via

**Affiliations:** 1 Faculty of Medicine and Surgery, University of Enna Kore, 94100 Enna, Italy; 2 Department of Medical and Surgical Sciences and Advanced Technologies “GF Ingrassia” ENT Section, University of Catania, 95123 Catania, Italyclaudio@claudiovicini.com (C.V.); 3 Head-Neck and Oral Surgery Unit, Department of Head-Neck Surgery, Otolaryngology, Morgagni Pierantoni Hospital, 47121 Forlì, Italy; giovanni.cammaroto@hotmail.com; 4 Department of Anesthesiology and Intensive Care, AOU “Policlinico—San Marco”, 95123 Catania, Italy; luigilavia7@gmail.com

We recently read with interest the study by Kuik et al. examining patient-reported outcomes following maxillomandibular advancement (MMA) surgery for obstructive sleep apnea (OSA) [1]. This research underscores the importance of integrating subjective assessments into OSA treatment investigations.

As the authors astutely noted, previous MMA research has predominantly centered on objective metrics, such as the apnea–hypopnea index (AHI) [2,3]. However, a singular focus on the AHI may inadvertently sideline outcomes that carry substantial weight for patients, like symptom burden and quality of life. Kuik et al.’s study counters this trend by evaluating post-MMA outcomes across various domains, including sleepiness, sleep quality, mandibular function, and general health. Doing so provides a more comprehensive evaluation rooted in patients’ lived experiences.

The findings of this study—improved sleepiness and quality of life but worsened mandibular function—warrant further investigation. Specifically, the observed decline in mandibular function could have long-term implications for oral health, necessitating attention. This potential disadvantage of MMA surgery should be clearly articulated during informed consent discussions, enabling patients to make an informed decision by balancing the risks and benefits.

Regarding the methodology, the application of validated questionnaires across different domains is commendable. However, the study’s single-center design and retrospective polysomnography (PSG) data collection may limit the generalizability of the findings and data quality. Future research could benefit from large-scale, prospective, multi-center studies that track patient-reported measures from the point of OSA diagnosis to better understand how treatments impact quality of life over time.

In summary, Kuik et al.’s study underscores the importance of patient-centered research in evaluating OSA treatments. Utilizing well-designed, patient-focused studies that explore the multifaceted impact of OSA and its treatments on patients’ lives should become standard practice [4,5]. This shift in the research paradigm is necessary to better inform clinical decision-making and ensure treatments holistically enhance patients’ well-being.

Integrating patient voices is crucial for guiding clinical decisions and ensuring therapies holistically enhance quality of life [6]. Kuik et al.’s study offers a valuable blueprint for the patient-focused research needed to optimize OSA treatment and improve patients’ well-being comprehensively and meaningfully.
